# Supplementary material for: Conferring the Metabolic Self-Sufficiency of the CAM Plasmid of Pseudomonas putida ATCC 17453: The Key Role of Putidaredoxin Reductase
Source: Microorganisms. 2019 Sep 26;7(10):395. doi: 10.3390/microorganisms7100395 (PMC6843404; doi:10.3390/microorganisms7100395)
Supplement: Supplementary file 1 [file microorganisms-07-00395-s001.pdf]

microorganisms

MDPI

Supplementary Materials:

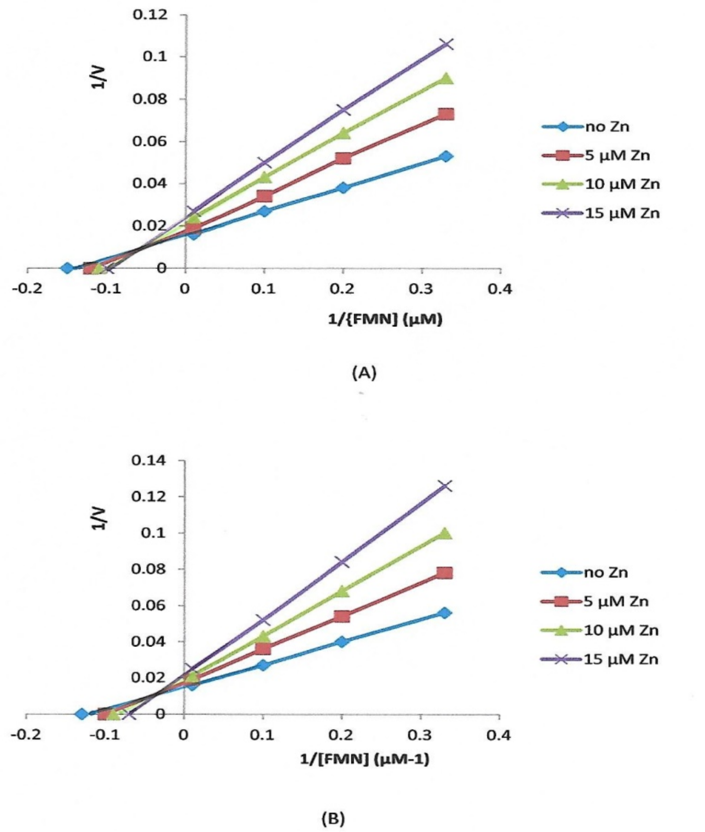

Figure S1. (**A**) Frp1 initial velocity as a function of NADH in the presence of 3.0, 5.0, 10.0 and 100.0 µM FMN in the absence of Zn^{2+} and with the divalent metal ion added to concentrations of 5.0, 10.0 and 15.0 µM. (**B**) Equivalent plots for Frp2

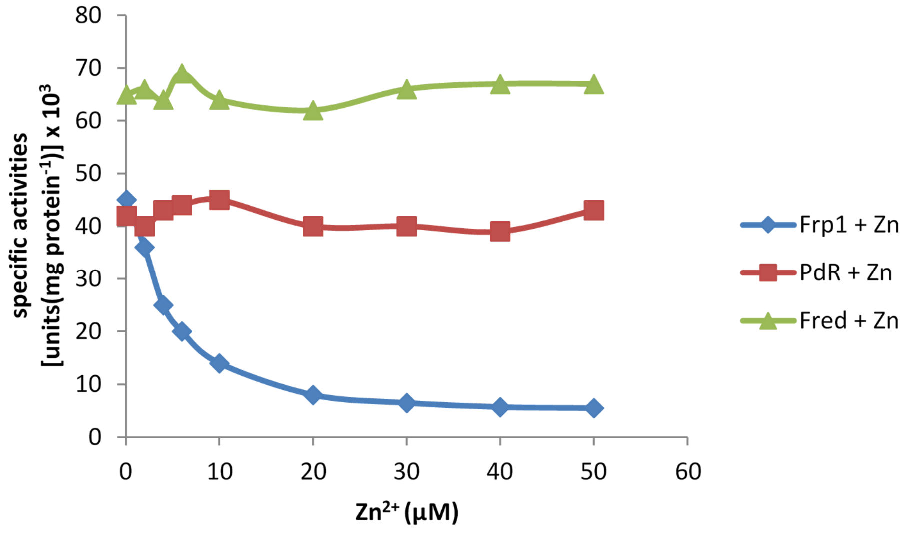

Figure S2. Activity of highly purified preparations of PdR and Fred in the presence of increasing levels of Zn^{2+}. Equivalent data for highly purified Frp1 is included for comparative purposes.

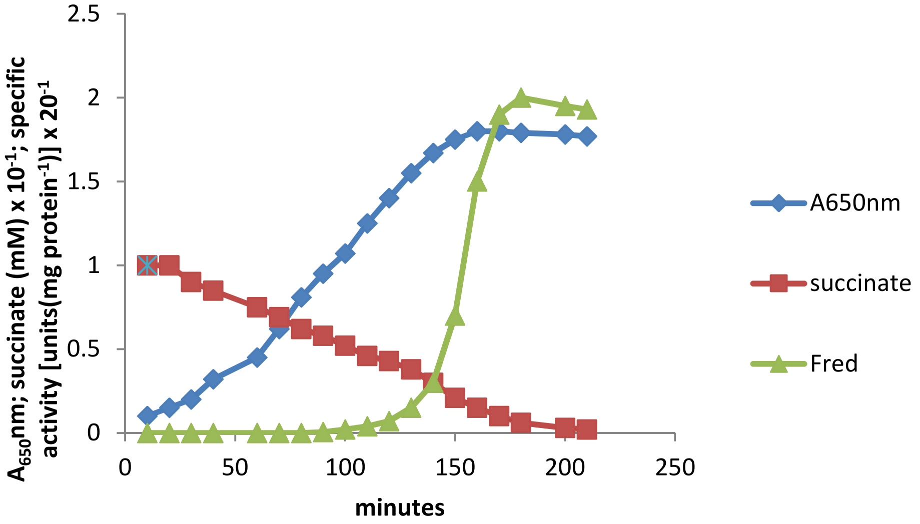

early | mid | late | stationary/
log log log idiophase

Figure S3. Growth of *P. putida* ATCC 17453 on 10 mM succinate minimal medium and the induction profile of Fred.
